# Salivary periostin levels as a non-invasive biomarker and their clinical correlates among healthy and periodontitis patients—a cross-sectional analytical study

**DOI:** 10.3389/fdmed.2025.1512252

**Published:** 2025-03-19

**Authors:** Priyanka Padalkar, Sunaina Shetty Yadadi, Gopinath Vivekanandan, Shishir Ram Shetty, Mangesh Andhare, Aditi Pashine, Vineet Vinay, Vijay Desai, Raghavendra M. Shetty

**Affiliations:** ^1^ Department of Periodontology, Aditya Dental College, Beed, India; ^2^ Department of Preventive and Restorative Dentistry, College of Dental Medicine, University of Sharjah, Sharjah, United Arab Emirates; ^3^ Department of Periodontology, Vivekanandha Dental College for Women, Tiruchengodu, India; ^4^ Department of Oral and Craniofacial Health Sciences, College of Dental Medicine, University of Sharjah, Sharjah, United Arab Emirates; ^5^ Associate Dentist, MyDentist, Hungerford, United Kingdom; ^6^ Department of Public Health Dentistry, Sinhgad Dental College and Hospital, Pune, India; ^7^ Department of Clinical Sciences, College of Dentistry, Ajman University, Ajman, United Arab Emirates; ^8^ Center of Medical and Bio-allied Health Sciences Research, Ajman University, Ajman, United Arab Emirates; ^9^ Department of Pediatric and Preventive Dentistry, Sharad Pawar Dental College and Hospital, Datta Meghe Institute of Higher Education and Research, Wardha, India

**Keywords:** biomarker, periodontitis, periostin, saliva, clinical attachment loss, salivary periostin

## Abstract

**Background:**

The diagnosis of periodontitis is primarily through clinical and radiographic assessments. However, it is difficult for clinicians to detect incipient periodontitis during the routine clinical assessment. Identifying people at risk for periodontitis and tracking disease development need a dependable biomarker. Currently, no biomarkers meet all the criteria required for an ideal diagnostic test. Therefore, the clinical utility of salivary periostin as a potential screening tool for periodontitis warrants further investigation, particularly through large samples across diverse populations. The present study aimed to investigate salivary periostin levels as a biomarker in individuals with periodontitis and healthy controls.

**Methods:**

Forty-five patients with generalized periodontitis stage III grade A/B and an equivalent number of periodontally healthy controls were evaluated for plaque index (PI), gingival index (GI), pocket probing depth (PPD), and clinical attachment level (CAL). Unstimulated salivary samples from all subjects were taken, and periostin levels were quantified using an ELISA kit.

**Results:**

The average salivary periostin levels were 4.63 in the healthy group and 1.24 in the periodontitis group (*P* < 0.05). The Spearman coefficient indicated a negative correlation between periostin levels and the gingival index (*r* = −0.761), plaque index (*r* = −0.780; *P* < 0.05), probing pocket depth (PPD) (*r* = −0.713; *P* < 0.05) and clinical attachment level (CAL) (*r* = −0.713; *P* < 0.05). Linear regression analysis validated the indirect correlation between salivary periostin levels and clinical indicators (Adjusted R square = 0.947).

**Conclusions:**

Salivary periostin levels are associated with periodontal disease. Salivary periostin levels indirectly influence as a non-invasive biomarker of periodontitis. The biomarker periostin is effective for evaluating both healthy and diseased periodontium.

## 1 Introduction

“Prospective healthcare” is an innovative methodology that evaluates an individual's health condition and vulnerability to the potential onset of clinical issues (1–3). The efficient implementation of resources to prevent diseases is crucial for cost-effective healthcare strategies, particularly in middle and low-income nations. Reliable and economical healthcare services can be achieved by advancing diagnostic methods to identify and classify at-risk patients (4). Recent research on molecular biomarkers like genomic biomarkers, transcriptomic biomarkers, proteomic biomarkers and metabolic biomarkers aims to identify quantifiable indicators of physiological, pathological, pharmacological, or genetic processes, which can be utilized to predict diagnosis and prognosis.

Human saliva, being readily accessible, possesses a composite nature and dynamic content, rendering it a viable medium for biomarker detection. It comprises proteins, nucleic acids, lipids, and metabolites that may be associated with oral and systemic diseases (5). Moreover, utilizing salivary fluids for disease activity recognition presents the significant benefits of being cost-effective, quick, and non-invasive. The combined application of modern technologies such as proteomics, metabolomics, lipidomics, and microbiomics has resulted in the development of a novel diagnostic approach termed “salivaomics” (5–9).

Periodontal diseases are significant among oral illnesses due to their elevated prevalence rates. Moreover, these diseases serve as a shared risk factor for various systemic diseases or conditions, including cardiovascular disease, pre-term low birth weight, Type 2 Diabetes Mellitus, multiple sclerosis, lupus erythematosus, oral cancer, polycystic ovarian syndrome, and negative outcomes in COVID-19 patients (10–16). Currently, clinical characteristics serve as conventional methods for identifying and diagnosing periodontitis.

The mechanisms linking periodontitis to other systemic diseases align with clinical findings that associate periodontitis with bacteremia, chronic low-grade inflammation, increased myelopoietic activity, and the effectiveness of local periodontal treatments in reducing systemic inflammation and improving disease activity markers (17–19).

On the other hand, systemic conditions like type 2 diabetes (T2DM) may increase the risk of developing periodontitis by heightening the inflammatory load on the periodontium or altering the periodontal microbiome (17, 20, 21).

The variability of clinical measures in evaluating the onset of risk factors for periodontitis underscores the urgent necessity for dependable disease biomarkers. The development of periodontitis biomarkers is a paramount priority that could transform the prevention and treatment of this condition.

Periostin is a distinctive proteomic biomarker that not only aids in diagnosing disease but also indicates severity, monitors treatment response, and forecasts prognosis in conditions such as cardiac repair, bronchial asthma, and type 2 diabetes mellitus (22–25). “Periostin” is a novel biomarker that plays a key role in processes such as collagen synthesis, cell migration, wound healing, and the regulation of periodontal pathogenesis (25–33). Nevertheless, experimental animal studies provided most of the information regarding salivary periostin levels and periodontitis circumstances compared to human studies (28–33), prompting more human research for external validity. Though there are sufficient studies on the periostin level in gingival crevicular fluid (GCF), the research on the periostin level in saliva is scanty. Hence, the present study aimed to assess salivary periostin levels as a non-invasive biomarker and to correlate with the clinical parameters among healthy and periodontitis patients.

## 2 Materials and methods

### 2.1 Study design and ethical approval

**Figure 1:**
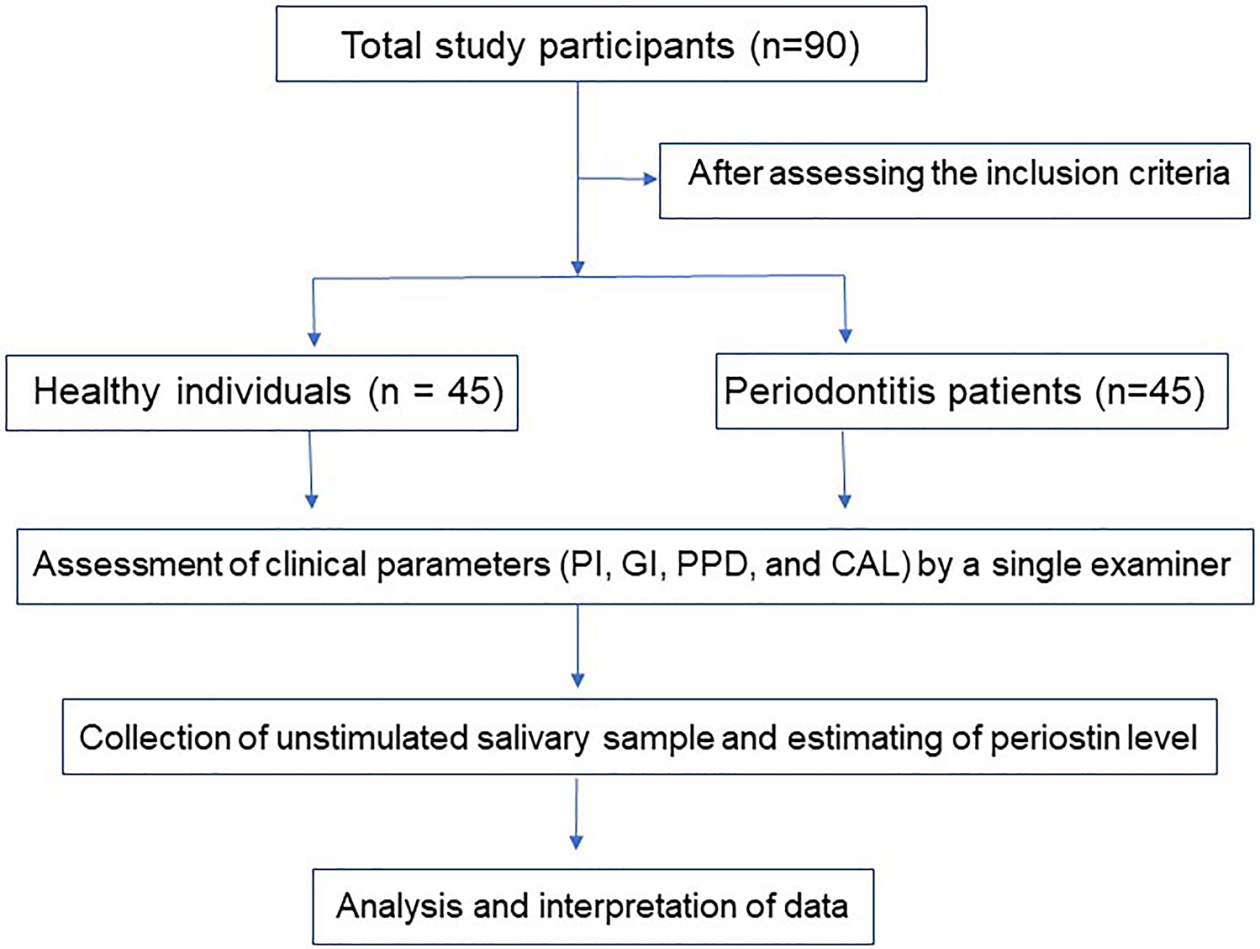
Flowchart of the study.

An Analytical Cross-Sectional study was designed, and ethical approval was secured from the Institutional Ethical Committee of the University of Health Sciences Nashik (SYN/PERIO/ADC/BEED/2462/2018). This research adhered to the “Strengthening the Reporting of Observational Studies in Epidemiology” (STROBE) criteria (34). Figure 1 depicts the flowchart of the investigation.

### 2.2 Selection of patients

Participants attending the outpatient Department of Periodontology at Aditya Dental College in Beed, Maharashtra, India, enrolled in this analytical cross-sectional study. The primary author elucidated the technique to all participants. Upon obtaining written informed consent from the volunteered participants, they signed up for the study.

The control group (Group I) comprised healthy patients exhibiting bleeding on probing of less than 10% and probing pocket depth (PPD) of less than 4 mm. The case group (Group II) comprised periodontitis patients aged 30 years and older, possessing at least 2 non-adjacent teeth with probing pocket depth (PPD) ≥ 5 mm, interdental clinical attachment loss (CAL) ≥ 5 mm, positive bleeding on probing, and radiographic evidence of alveolar bone loss classified as generalized periodontitis Stage III Grade B. The participants were categorized as having generalized periodontitis stage III grade A/B or as healthy individuals following a comprehensive intraoral examination per the latest classification of periodontal and peri-implant diseases 2017 (35–37). Individuals who habitually used tobacco in any form, pregnant women, those with a history of systemic disease, individuals who have used antibiotics within the past six months, and those who have received any sort of periodontal treatment during the last two years were excluded from the study.

### 2.3 Assessment of study participants' parameters

To ascertain the sample size for our study, we utilized the standard normal deviates for Type I error (α) and Type II error (β), denoted as Zα and Zβ, respectively. In our analysis, Zα is established at 1.9600 and Zβ at 0.8416. We computed the coefficient C, defined as 0.5 * ln[(1 + r)/(1-r)], where r denotes the correlation coefficient (0.461) (23). We determined the overall sample size (N) by the formula *N* = [(Zα+Zβ)/C]^2^ + 3. This necessitates a sample size of 35 persons. We included 45 people in each group. This calculation validates that our investigation possesses adequate statistical power to identify the stated effects at a 95% confidence range and 80% power.

This study recruited ninety individuals, comprising an equal number (*n* = 45) of subjects with periodontitis and healthy controls. Participants' gingival status and periodontal condition were evaluated using established procedures based on Plaque Index scores, Pocket Probing Depth scores (PPD), Gingival Index scores, and Clinical Attachment Level (CAL). Intra-examiner reliability was assessed using Kappa statistics, yielding a value of 0.75. The four regions were documented for the Plaque Index and Gingival Index (38, 39). The distance from the coronal free gingival border to the base of the pocket for each tooth was assessed using the Williams periodontal probe (Hu Friedy, Chicago, IL, USA). The deepest pocket recorded was deemed a PPD score for the specific subject (40). The Clinical Attachment Level (CAL) was measured from the cementoenamel junction to the base of the gingival sulcus/periodontal pocket using a periodontal probe at all six designated sites for probing depth (41). Only the primary author evaluated all patients' PPD and CAL scores to ensure consistency in proprioception assessment. The radiographic examination was conducted to corroborate the clinical diagnosis of widespread periodontitis stage III grade A/B in Group II and to assess the health status of patients in Group I.

### 2.4 Assessment of salivary periostin concentration

Participants were instructed to abstain from consuming food or beverages for a minimum of 2 h before the collection of salivary samples. Unstimulated saliva samples were obtained from all the participants (42, 43). Samples were obtained between 9 a.m. and 12 p.m. to mitigate diurnal oscillations coinciding with the periods, individuals attended the outpatient department. Each volunteer gargled with water approximately 10 min before the initiation of salivary collection. Subsequently, participants were seated on the dental chair, and their saliva was collected passively into a labeled sterile container by instructing them to expectorate until the volume reached 5 ml. All samples underwent centrifugation for 5 min to eliminate turbidity and cellular debris. The samples were preserved at −80°C in Eppendorf tubes until subsequent processing. Following thawing, the periostin concentration in preserved samples was quantified utilizing an ELISA kit and administered following the manufacturer's guidelines (RD191016100; BioVendor Laboratory Medicine, Brno, Czech Republic). The ELISA plate reader (BioTeK Instruments Inc., Winooski, VT, USA) was employed to quantify periostin levels by correlating them with absorbance at a wavelength of 450 nm. The periostin level was expressed as nanograms per milliliter (ng/ml) with a 1–50 ng/ml detection range of the ELISA kit.

### 2.5 Statistical analysis

The Statistical Package for the Social Sciences (SPSS) software version 21 (SPSS Inc., Chicago, IL, USA) was utilized to analyze the descriptive and inferential statistics of the results. Due to the Kolmogorov–Smirnov test indicating non-normality, the Mann–Whitney *U*-test was employed to evaluate GI, PI, PPD, CAL, and periostin levels between healthy individuals and patients with periodontitis. A *P*-value less than 0.05 was deemed statistically significant. The Spearman correlation test evaluated the association between salivary periostin and GI, PI, PPD, and CAL. Additionally, a regression analysis was carried out to assess the impact of independent variables on salivary periostin levels. Receiver-operating characteristic curve (ROC) was carried out to assess the diagnostic accuracy and the area under the curve (AUC) was generated.

## 3 Results

Ninety volunteers participated in the current investigation. Group I included 45 individuals, 30 males and 15 females, whereas Group II also comprised 45 individuals, 27 males and 18 females. No significant difference was seen in the distribution of research participants considering gender and age group (Table 1).

**Table 1:** Distribution of study participants according to gender and age groups.

	Group I *n* (%)	Group II *n* (%)	Significance^#^	
			*χ*^2^ value	*P*-value
Gender				
Male	30 (66.67)	27 (60)	0.43	0.51
Female	15 (33.33)	18 (40)		
Age groups (years)				
30–40	15 (33.33)	11 (24.44)	1.01	0.60
41–50	19 (42.22)	20 (44.44)		
51–60	11 (24.44)	14 (31.11)		

^#^ Chi square test.

All subjects exhibited the presence of periostin in their salivary samples. The average salivary periostin levels in Group I and Group II were 4.63 and 1.24 ng/ml, respectively, indicating a substantial disparity between the groups in our study. The evaluated clinical indicators demonstrated a statistically significant difference between Group I and Group II in gingival index scores, plaque index scores, pocket probing depth, clinical attachment loss, and salivary periostin levels (Table 2; *P* < 0.001).

**Table 2:** Intergroup comparison of gingival index, plaque index, pocket probing depth, clinical attachment level and salivary periostin levels.

Parameters	Group	*N*	Mean	Std. Deviation	*P*-value^#^
PI	Group I	45	0.902	0.122	0.000**
	Group II	45	1.695	0.193	
GI	Group I	45	0.841	0.092	0.000**
	Group II	45	1.308	0.204	
PPD (mm)	Group I	45	2.9556	3.096	0.000**
	Group II	45	6.9333	0.889	
CAL (mm)	Group I	45	2.4889	0.505	0.000**
	Group II	45	8.9556	0.877	
SPL (ng/ml)	Group I	45	4.6398	35.893	0.000**
	Group II	45	1.2427	13.788	

GI, gingival index; PI, plaque index; PPD, pocket probing depth; CAL, clinical attachment level; SPL, salivary periostin levels.

^#^ Mann–Whitney *U*-test.

** Highly Significant (*P* < 0.001).

The Spearman coefficient indicated a negative correlation between periostin levels and the gingival index (*r* = −0.761; *P* < 0.001), plaque index (*r* = −0.780; *P* < 0.001), probing pocket depth (PPD) (*r* = −0.713; *P* < 0.001), and clinical attachment level (CAL) (*r* = −0.713; *P* < 0.001) (Table 3). Linear regression analysis further elucidated the robustness of the indirect correlation between salivary periostin levels and the clinical measures employed in the study (Table 4).

**Table 3:** Correlation between salivary periostin and various periodontal clinical parameters.

Parameter	Statistics	GI	PI	PPD	CAL
Salivary Periostin Level (SPL)	Correlation Coefficient	−0.761	−0.780	−0.713	−0.785
	Sig. (2-tailed)^$^	.000**	.000**	.000**	.000**

^$^ Spearmans Rho; GI, gingival index; PI, plaque index; PPD, pocket probing depth; CAL, clinical attachment level.

**Highly significant (*P* < 0.001).

**Table 4:** Multivariate regression analysis of salivary periostin levels with gingival Index, plaque index, pocket probing depth, clinical attachment level scores.

Parameters	Unstandardized Coefficients		Standardized Coefficients	Adjusted R Square	Significance	
	B	Std. Error	Beta		*t*	*P*-value
GI	−111.297	23.023	−0.277	0.947	−4.834	0.000**
PI	−27.107	27.189	−0.044	0.947	−0.997	0.032*
PPD	4.262	1.917	0.074	0.947	2.223	0.029*
CAL	−38.006	3.158	−0.732	0.947	−12.035	0.000**

GI, gingival index; PI, plaque index; PPD, pocket probing depth; CAL, clinical attachment level.

* Significant (*P* < 0.05).

** Highly significant (*P* < 0.001).

**Figure 2:**
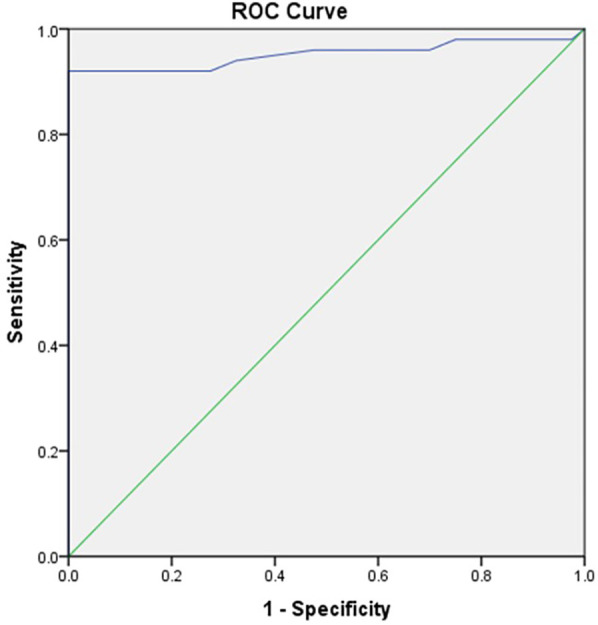
Receiver operating characteristics curve (ROC) depicting area under the curve (AUC) for salivary periostin level (ng/ml).

ROC analysis revealed the area under the curve (AUC) value of 0.952, which indicated excellent diagnostic ability. AUC values range from 0.5 (no discrimination) to 1.0 (perfect discrimination). An AUC of 0.952 suggested that the test variable (salivary periostin level) can distinguish between the positive and negative groups. The *P*-value was less than 0.001, which was highly significant. This suggested that the observed AUC significantly differs from the null hypothesis value (AUC = 0.5), indicating strong evidence that the test variable is predictive (Figure 2).

## 4 Discussion

The periodontium, a highly specialized tissue, supports, protects, and enables proprioception within the oral cavity. This tissue consists of various cells vital for maintaining the integrity of the teeth' structures (43–47). Periodontal disease, which encompasses a broad spectrum of clinical manifestations, can range from mild symptoms, such as gingival bleeding, to more severe forms that result in attachment loss, bone degradation, tooth mobility, and eventual tooth loss. The progression of this disease can have profound consequences on oral health and overall well-being. Epidemiological data suggest that periodontal disease affects approximately 20%–50% of the global population, positioning it as one of the most prevalent oral health issues worldwide. It is ranked as the ninth most common illness-related impairment, impacting millions of individuals across different regions and populations (48, 49).

Periodontal disease arises from intricate interactions between the host immune response and subgingival biofilms. While everyone reacts to this immunomodulation differently, the disease can generally be averted in most cases (50). Current clinical diagnostic measures can only assess prior episodes of tissue destruction, specifically the threshold shift in periodontal cells, which may indicate 2–3 mm of tissue destruction before clinicians can detect a current disease site. Advanced diagnostic tools, such as subtraction radiography, are accessible for diagnosing periodontal disease. Nonetheless, dental care providers' routine application, cost-effectiveness, and patient acceptance results in their underutilization (4).

The pathogenesis of periodontal disease involves a complex interplay between the host's immune response and subgingival biofilms, which harbor a variety of pathogenic microorganisms. While the immune response to these microorganisms varies among individuals, periodontal disease is largely preventable if proper preventive measures are taken (50). However, current diagnostic tools primarily focus on detecting tissue damage after it has already occurred, typically when there is already a significant loss of periodontal tissue, such as a 2–3 mm shift in attachment levels, which may not be identified until substantial destruction has taken place. While useful, conventional diagnostic methods, such as probing and radiographic assessments, are often limited by their inability to detect early disease stages and their reliance on visible or measurable tissue destruction. Although advanced diagnostic techniques, such as subtraction radiography, show promise for detecting periodontal disease in its early stages, their high cost and limited routine application by dental care providers often lead to underutilization that highlights a significant gap in clinical practice and a need for alternative, cost-effective methods for early detection. Therefore, a viable new diagnostic approach that transitions from a “Disease-Centric” model of periodontal care to a “Patient-Centric” model, which evaluates individual patient risk factors, will significantly interest healthcare professionals (50).

A shift towards a more patient-centric approach in periodontal care, where individual risk factors and molecular markers are considered, offers great potential for improving diagnosis and treatment. This personalized approach could provide more targeted, effective interventions and better align with individuals' diverse responses to periodontal disease. Recent advances in “omics” technologies have introduced the possibility of utilizing molecular markers, such as those found in saliva, to predict an individual's predisposition to periodontal disease. These biomarkers are particularly advantageous as they are non-invasive, easily accessible, and can provide real-time insights into an individual's periodontal health.

Castagnola et al. (51) indicated that holistic technologies incorporating omics sciences in saliva could, shortly, accurately predict the complex individuality of each individual by leveraging their distinct traits such as fingerprints and the molecular mechanisms associated with health and disease, and vice versa (51). Periostin has emerged as a promising biomarker in periodontal disease among the various molecular markers. Periostin is a secreted extracellular matrix protein predominantly expressed in periodontal ligaments, where it plays a crucial role in tissue integrity, bone remodeling, and tooth development (52–58). This protein has been identified in several biological fluids, including saliva, where its presence and concentration could potentially indicate periodontal health. The present study involved 90 volunteers divided equally into two groups, Group I and Group II. There were no significant differences in gender or age distribution, which ensured that the results were not biased by these demographic factors. All participants had detectable levels of periostin in their salivary samples, confirming the presence of this biomarker across both groups.

A notable finding of this study is the substantial difference in average salivary periostin levels between the two groups, with Group I exhibiting significantly higher levels (4.63) compared to Group II (1.24). This suggests a potential link between periostin levels, and the clinical condition being investigated, as indicated by the substantial difference in clinical measures between the two groups. Specifically, Group II showed significantly worse clinical indicators, including higher gingival index scores, plaque index scores, probing pocket depth (PPD), and clinical attachment loss (CAL), all of which are consistent with poorer periodontal health.

The correlation analysis further strengthens this relationship, revealing significant negative correlations between salivary periostin levels and clinical measures such as the gingival index (*r* = −0.761), plaque index (*r* = −0.780), probing pocket depth (*r* = −0.713), and clinical attachment level (*r* = −0.713), all with *P*-values less than 0.001. These findings suggest that as periostin levels decrease, the clinical indicators of periodontal disease worsen, which aligns with the study of Du and Li (43) indicating that periostin, a matrix protein involved in tissue remodeling, may play a role in the progression of periodontal disease (43).

Linear regression analysis further supported the strength and consistency of this negative correlation, reinforcing the potential utility of salivary periostin levels as an indirect marker of periodontal health. This is particularly significant as it offers a non-invasive method for monitoring periodontal disease progression, which could benefit clinical settings.

The receiver operating characteristic (ROC) analysis also demonstrated an excellent diagnostic ability for salivary periostin, with an area under the curve (AUC) value of 0.952. This high AUC indicates that salivary periostin levels can effectively differentiate between individuals with differing clinical presentations of periodontal health and disease. The AUC value, significantly higher than the null hypothesis value of 0.5, supports the strong predictive value of salivary periostin levels for periodontal disease.

Our investigation found no statistically significant variations in mean age between the control and experimental groups, unlike the findings of Jamesha et al. (50), since the age of Group II in the experimental group was higher than that of the control group (50). Substantial data indicates increased plaque build-up and elevated gingival index scores in people with periodontitis (23, 33, 50, 59, 60) corroborating with the present study.

The results of our investigation indicated that periostin expression in saliva was markedly decreased in patients with periodontitis. In accordance with the current study, research conducted by Aral et al. (33) that measured periostin levels in saliva and gingival crevicular fluid (GCF) in periodontitis patients revealed lower values (33). Similarly, Esfahrood et al. (61) observed findings consistent with the current study in their focused research on salivary periostin and chronic periodontitis (34). The only difference was that the latest 2017 classification of periodontal and peri-implant diseases was used to diagnose periodontitis in the present study. These findings may be attributed to periodontal inflammation, which could impair fibroblast development from totipotent cells, perhaps resulting in less periostin expression.

The findings of our study also indicated that periostin levels in saliva are significantly lower in individuals with periodontitis. This result aligns with studies by Aral et al. (33) and Esfahrood et al. (61), who found decreased periostin levels in the saliva and gingival crevicular fluid (GCF) of periodontitis patients (33, 34). These findings suggest that periodontal inflammation, which impairs the function of fibroblasts in the periodontal ligament, may lead to reduced periostin expression. Chronic inflammation in the periodontium can hinder the expression of periostin, thus contributing to the observed decrease in its levels in saliva and GCF. Research on periostin levels in the gingival crevicular fluid (GCF) of patients with chronic periodontitis indicated reduced periostin levels in these individuals (23, 50, 59, 62–64).

In the recent systematic review and meta-analysis by Abdolalian et al. (65), it was reported that the mean concentration of periostin in GCF is significantly lower in individuals with chronic periodontitis compared to those with gingivitis and healthy individuals (65). These results indicate that periostin may serve as a potential diagnostic marker for chronic periodontitis. Our findings are consistent with these results, although, in our study, we evaluated salivary periostin levels rather than GCF periostin.

In the present study, we found a clear indirect correlation between the presence of periodontitis and alterations in salivary periostin levels, further solidifying periostin's potential as a reliable marker for periodontal tissue inflammation. This non-invasive biomarker could offer several advantages over traditional diagnostic methods, including ease of collection, cost-effectiveness, and detecting early signs of disease before more significant tissue damage occurs. While another study by Aral et al. (33) has also corroborated the effectiveness of salivary periostin as a periodontal biomarker, our findings contribute to the growing body of evidence supporting its use as a practical tool for assessing periodontal conditions (33). Given the emerging importance of molecular biomarkers in modern periodontal diagnostics, salivary periostin holds promise for enhancing the early detection, management, and monitoring of periodontal disease, ultimately improving patient outcomes and progressing toward patient-centered care.

The results of the present study provide strong evidence supporting the potential of salivary periostin as a reliable biomarker for assessing periodontal health. The significant negative correlations with clinical measures of periodontal disease and the excellent diagnostic accuracy shown in the ROC analysis suggest that salivary periostin could be an important tool in the early detection and monitoring of periodontal disease. Further studies are warranted to explore its use in clinical practice, including its potential for early intervention and personalized treatment strategies in periodontal care.

### 4.1 Strengths, limitations, recommendations and future scope

The current investigation employed both subjective and objective approaches to verify the disease state. To reduce observer bias, a single examiner documented all the clinical parameters. Although the sample size is limited, our study offers significant insights into interpreting the population parameter of the dependent variable examined. Salivary periostin holds significant promise for the early detection and management of periodontitis. Through non-invasive tests, it can identify early signs of the disease even before clinical symptoms manifest. Salivary periostin biomarkers could be integrated into diagnostic devices that facilitate chair-side investigations in dental clinics during routine screenings. Once detected, these biomarkers can assist in monitoring disease progression, evaluating treatment effectiveness, and preventing relapses. Additionally, combining these biomarkers with clinical parameters can enhance the precision of periodontitis management, leading to more personalized and effective care for patients.

The present study did not evaluate the relationship between periostin levels and the severity of periodontal disease, which is a limitation. Future research that includes all stages of periodontitis as defined by the latest classification will provide a more complete understanding.Additional multicentric research worldwide is necessary to validate external applicability. The evaluation of the whole biochemical expression of this complex salivary protein by several diagnostic techniques, such as ELISA, quantitative real-time PCR, and mRNA analysis, is necessary. It is necessary to design more clinical trials to evaluate salivary periostin levels to validate this novel biomarker in identifying disease status and measuring its efficacy as a diagnostic tool post-treatment. Periodontitis is a chronic immunomodulatory disease that is influenced by several hormonal and systemic factors. Therefore, exploring fresh perspectives about periostin salivary levels in different periodontal disorders is advisable. Further studies need to be designed to evaluate the impact of systemic conditions/diseases on periostin levels that could confound the diagnosis of periodontitis and could benefit targeted screening of high-risk groups with systemic diseases and periodontitis.

## 5 Conclusions

Salivary periostin levels are associated with the periodontal disease. The periostin level was higher in healthy individuals when compared to the individuals with periodontal diseases included in the study. The study revealed significant negative correlations between salivary periostin levels and clinical measures such as the gingival index, plaque index, probing pocket depth, and clinical attachment level. Salivary periostin levels indirectly influence as a non-invasive biomarker of periodontitis. The biomarker periostin is effective for evaluating both healthy and diseased periodontal tissue.

The present study contributes new insights into the role of salivary periostin as a biomarker for periodontitis, which is evaluated within the framework of the latest classification system of the “2017 World Workshop classification of periodontal and peri-implant diseases and conditions” and marks a significant step forward in periodontal research. The non-invasive approach validates the relevance of periostin as a biomarker and supports the growing trend toward using biomarkers for the early detection and management of periodontal diseases. As more studies explore the relationship between salivary periostin and periodontal health, this biomarker will likely play an increasingly central role in clinical practice, enhancing the ability to monitor disease progression and response to treatment.

## Data Availability

The raw data supporting the conclusions of this article will be made available by the authors, without undue reservation.
